# Prediction of the therapeutic mechanism of Sugemule-4 in insomnia treatment using network pharmacology and molecular docking

**DOI:** 10.1097/MD.0000000000046489

**Published:** 2026-05-12

**Authors:** Rina Su, Jinjing Wang, Dena Su, Riguga Su, Chula Sa, Lechaolu Bao

**Affiliations:** a Mongolian Medical College, Inner Mongolia Medical University, Hohhot, China; b Medicine Innovation Center for Nationalities, Inner Mongolia Medical University, Hohhot, China; c Key Laboratory of Mongolian Medicine in Universities of Inner Mongolia Autonomous Region, Hohhot, China.

**Keywords:** insomnia, molecular docking, molecular dynamics simulation, Mongolian medicine, network pharmacology, Sugemule-4 (SGML-4)

## Abstract

This study aimed to identify the active components and related target pathways as well as examine the potential mechanisms of action of Sugemule-4 (SGML-4) for the treatment of insomnia, based on network pharmacology, molecular docking analysis, and molecular dynamics simulation. The active compounds of SGML-4 were retrieved from the Traditional Chinese Medicine Systems Pharmacology, Herbal Medicine Resource, and Encyclopedia of Traditional Chinese Medicine databases. Their potential targets were predicted using the SwissTargetPrediction platform, while known insomnia-related targets were gathered from GeneCards, Online Mendelian Inheritance in Man, Therapeutic Target Database, Drugbank, and PharmGKB. The intersection of compound-related targets was then determined. Next, a protein–protein interaction network was constructed and visualized using the STRING online platform and Cytoscape 3.10.3 software. Gene Ontology and Kyoto Encyclopedia of Genes and Genomes pathway enrichment analyses were conducted using the DAVID platform. Based on these analyses, key targets and the principal active compounds of Sugemule IV were selected for molecular docking studies using AutoDock software to evaluate their potential interactions. Finally, molecular dynamics simulations were conducted using GROMACS software to assess the stability of the protein–compound complexes. A total of 106 active compounds and 364 overlapping targets were identified. Luteolin, pinocembrin, piplartine, lysicamine, and apigenin showed the highest degree values, and AKT1, glyceraldehyde-3-phosphate dehydrogenase, tumor necrosis factor, albumin, and epidermal growth factor receptor (EGFR) were identified as core targets. Gene Ontology analysis indicated enrichment in chemical synaptic transmission and G protein-coupled receptor signaling. Kyoto Encyclopedia of Genes and Genomes analysis revealed serotonergic synapse and calcium signaling as major pathways. Molecular docking showed strong binding affinities between active compounds and targets, particularly EGFR, which formed stable hydrogen bonds. Molecular dynamics confirmed stable interactions of EGFR with apigenin, luteolin, and piplartine. SGML-4 exerts anti-insomnia effects through multi-target and multi-pathway mechanisms. Apigenin, luteolin, and piplartine are the core active components, and EGFR is identified as the central target, potentially acting through serotonergic synapses and calcium signaling pathways.

## Graphical abstract

**Figure:**
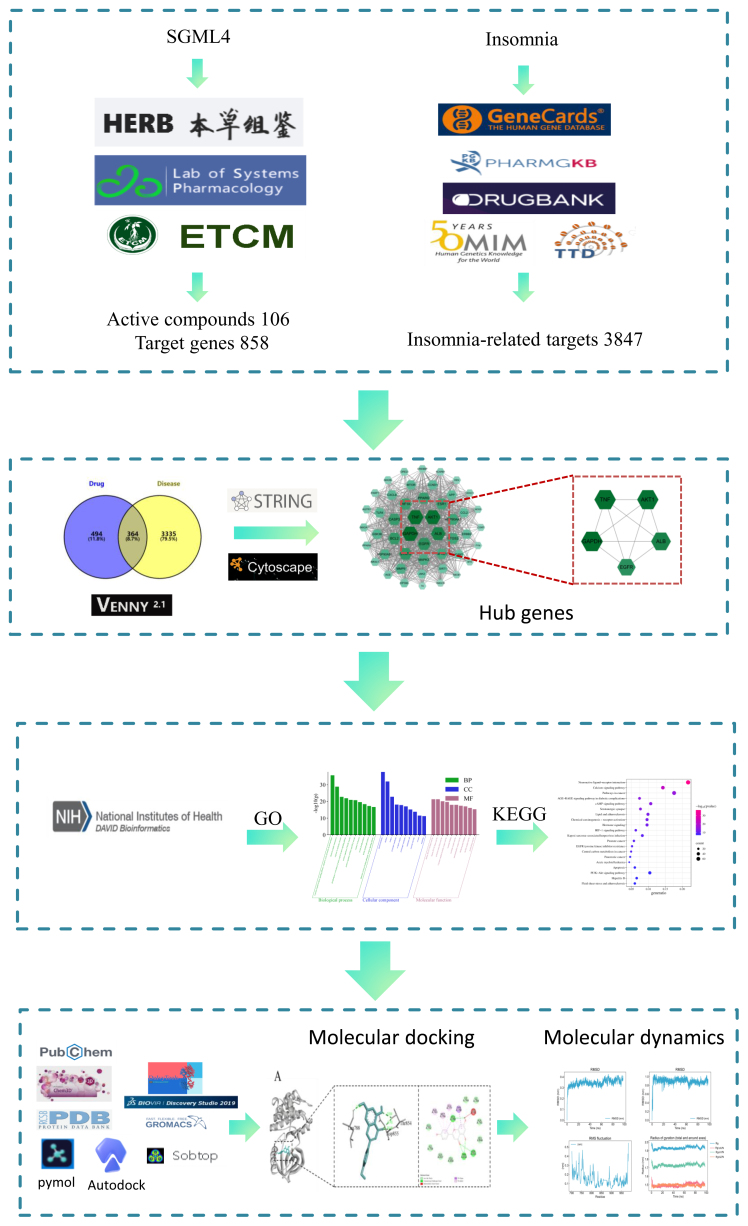

## 1. Introduction

Insomnia encompasses nocturnal symptoms, such as difficulty initiating sleep, difficulty maintaining sleep, and early awakening, and daytime symptoms, such as fatigue, impaired attention, mood abnormalities.^[1]^ The Diagnostic and Statistical Manual of Mental Disorders, Fifth Edition and the International Classification of Sleep Disorders, Third Edition classify insomnia as a separate disorder,^[2,3]^ rather than a secondary symptom of other conditions. Epidemiological studies indicate that approximately 10% to 30% of the global population experiences chronic insomnia symptoms, and in certain high-risk groups (e.g., older persons, females, shift workers, and individuals with psychiatric disorders), the prevalence exceeds 50%.^[4]^ The adverse effects of insomnia impact psychological health, cognitive and behavioral functioning, and physical health.^[5–7]^

Modern medicine contends that the pathophysiological mechanism of insomnia involves multiple factors, including neurotransmitter imbalance, hormonal dysregulation, and cognitive–behavioral elements.^[8–10]^ Clinical interventions include a combination of pharmacological and non-pharmacological treatments; however, their long-term use may lead to tolerance, dependence, and adverse side effects. Therefore, there has been an increasing focus on complementary and alternative medicine, which is grounded primarily on holistic theory and syndrome differentiation and has demonstrated promise for the treatment of insomnia.^[11]^ Mongolian medicine classifies insomnia under the disease category “Heyi.” Its etiologies are varied and may include excessive ruminating; having a heavy mental burden and prolonged sorrow; performing labor on an empty stomach; or consuming goat meat, peppercorns, and other bitter, light-natured, or coarse foods.^[12]^ Moreover, impaired circulation of Qi and blood, leading to stagnation, insufficient nourishment of the heart, or imbalance of visceral functions, may also contribute to the development of insomnia.^[13]^ Pharmacotherapy is the mainstay of Mongolian medical treatment for insomnia.^[14,15]^ Traditional Mongolian prescriptions, including Sugemule-3,^[16–18]^ Sugemule-4 (SGML-4),^[19]^ and Gaoyou-13,^[20]^ have demonstrated significant therapeutic efficacy in clinical practice. Notably, SGML-4 is a well-established formula created by Professor Su Rongzhabu, a recognized Master of Traditional Chinese Medicine in China, and has garnered significant attention. SGML-4 comprises 4 medicinal components: *Amomum kravanh*, *Piper longum*, *Ligusticum sinense*, and *Ziziphus jujuba* var. spinosa. It is recognized for its efficacy in suppressing “Heyi” and is specifically indicated for the treatment of insomnia, serving as a highly effective sedative.^[21]^ Recent studies of SGML-4 have primarily focused on its chemical composition, anti-insomnia effects, and underlying mechanisms. Guo et al^[22]^ found that the n-hexane and ethyl acetate extracts of SGML-4 mainly consist of terpenoids, triterpenoids, phenylpropanoids, and alkaloids. Doulina et al^[19]^ reported that SGML-4 decoction, its component formulas, and individual herbs could alleviate insomnia by regulating related neurotransmitters such as serotonin (5-HT) and gamma-aminobutyric acid (GABA). The alkaloids nuciferine and tetrahydroberberine contained in SGML-4 were shown to improve insomnia symptoms induced by 4-chloro-DL-phenylalanine (PCPA).^[23]^ Li et al^[24]^ demonstrated that the mechanism by which SGML-4 improves sleep duration and behavioral performance in insomnia model rats may be associated with the regulation of tryptophan and tyrosine metabolism pathways. Li et al^[25]^ further found that SGML-4 could ameliorate primary insomnia by modulating the P2X7 receptor and related signaling pathways. Existing studies have confirmed that SGML-4 contains multiple classes of compounds, including terpenoids and alkaloids, and have preliminarily revealed its anti-insomnia effects through modulation of neurotransmitters, amino acid metabolism, and the P2X7 receptor. However, the overall regulatory network linking “active components–core targets–key pathways” has not yet been systematically elucidated, and the molecular binding characteristics between key constituents such as nuciferine and insomnia-related targets remain insufficiently validated.

Emerging technologies such as network pharmacology, molecular docking, and molecular dynamics simulation have become important strategies for elucidating the mechanisms of traditional Chinese medicine and multi-component drugs in modern pharmacological research. In recent years, an increasing number of studies have applied systems pharmacology to predict the mechanisms of herbal medicines in the treatment of insomnia. For example, Shuxiao et al^[26]^ used network pharmacology and molecular docking to reveal that Suanzaoren Tang may exert therapeutic effects by regulating neurotransmitter function through pathways such as neuroactive ligand–receptor interaction and serotonergic synapse; activating the PI3K/AKT signaling pathway to inhibit neuronal apoptosis and inflammation; and reducing the levels of inflammatory cytokines such as IL-6 and tumor necrosis factor (TNF) to alleviate neuroinflammation. Liu et al^[27]^ integrated data mining, network pharmacology, GEO verification, and molecular docking analyses to explore the potential mechanisms of traditional Chinese medicine in treating insomnia. Their results indicated that the core active compounds (β-sitosterol, stigmasterol, and cannabinol) targeted key proteins including IL-6, CASP3, and TP53, thereby regulating inflammation, neural signaling pathways, and circadian rhythm, achieving a “multi-component–multi-target–multi-pathway” therapeutic effect. Lin et al.^[28]^ further revealed through systems pharmacology that Sinisan may alleviate anxiety-related insomnia by modulating neuroinflammation, regulating neurotransmitter transmission, and protecting neurons.

Therefore, this study employed network pharmacology, molecular docking, and molecular dynamics simulation to systematically integrate the chemical constituents and potential targets of SGML-4. The aim was to predict key pathways and core proteins associated with sleep regulation and to verify the binding affinities and interaction modes between candidate compounds and their receptors through molecular docking analysis. Meanwhile, molecular dynamics simulations were conducted to evaluate the stability and interaction mechanisms of compound–target complexes, thereby improving the reliability of the predictions. This integrated design enables a systematic elucidation of the multi-component, multi-target, and multi-pathway synergistic mechanisms underlying the anti-insomnia effects of SGML-4. Furthermore, it provides a theoretical foundation and precise molecular targets for subsequent in vitro and in vivo experimental validation.

## 2. Methods and materials

### 2.1. Screening of the active components and related targets of SGML-4

The components of the SGML-4 formula were sourced from the Traditional Chinese Medicine Systems Pharmacology database (https://old.tcmsp-e.com/tcmsp.php),^[29]^ the Herbal Medicine Resource database (http://herb.ac.cn/),^[30]^ and the Encyclopedia of Traditional Chinese Medicine database (http://www.tcmip.cn/ETCM/index.php/).^[31]^ The active components were screened using SwissADME (http://www.swissadme.ch/).^[32]^ These indices have been extensively used in pharmaceutical chemistry studies to evaluate the oral bioavailability and drug-likeness of candidate compounds.^[33]^ Furthermore, additional ADME parameters were taken into account to ensure that the selected compounds exhibited desirable pharmacokinetic properties in vivo. *Ligusticum sinense* was not included in the database; therefore, its chemical components were obtained from a literature search and then screened. After identifying the effective active components, potential targets were predicted using the SwissTargetPrediction platform (http://www.swisstargetprediction.ch/citing.php).^[34]^ A probability threshold of > 0.1 was applied for target selection to improve the credibility and accuracy of the prediction results.

### 2.2. Identification of disease targets and potential therapeutic targets of SGML-4 for insomnia

Disease-related targets were identified using the keyword “Insomnia” from the following disease-related databases: GeneCards (http://www.genecards.org/),^[35]^ Online Mendelian Inheritance in Man (http://omim.org/),^[36]^ Therapeutic Target Database (http://db.idrblab.net/ttd),^[37]^ DrugBank (https://go.drugbank.com/),^[38]^ and PharmGKB (https://www.pharmgkb.org/).^[39]^ The obtained targets were standardized through the UniProt database, with the species restricted to *Homo sapiens*, and only “reviewed” entries were retained. The predicted targets of the active compounds and the disease-related targets were then imported into Venny 2.1^[40]^ to identify their intersection, which represented the potential therapeutic targets of SGML-4 for insomnia. A Venn diagram was subsequently generated to visualize the overlapping targets.

### 2.3. Construction of a protein–protein interaction network for intersecting targets

The overlapping targets were uploaded to the STRING platform (https://string-db.org/)^[41]^ for protein–protein interaction (PPI) network analysis, with the species set as *Homo Sapiens*. During the construction of the interaction network, the minimum required interaction score was set to 0.4, corresponding to a medium confidence level. The resulting TSV file generated by STRING was imported into Cytoscape 3.10.3 (Cytoscape Consortium, San Diego) for visualization. Topological parameters, including betweenness centrality, closeness centrality, and degree centrality, were determined using the CytoNCA plugin in Cytoscape. Based on these parameters, the core targets were identified.

### 2.4. Gene ontology (GO) function enrichment analysis and Kyoto Encyclopedia of Genes and Genomes (KEGG) pathway enrichment analysis

GO function and KEGG pathway enrichment analyses were condcuted using the David database (https://david.ncifcrf.gov/).^[42,43]^ The GO categories included biological process (BP), cellular component (CC), and molecular function (MF). The enrichment results were sorted based on the GO terms, count values, and *P* values. Terms with a *P* value of < .05 were considered statistically significant.^[44]^ The top 10 GO terms and the top 10 KEGG pathways (ranked by *P* value) were selected for further analysis and visualized using the online platform Bioinformatics (http://www.bioinformatics.com.cn).^[45]^ To examine the interactions among the drugs, active compounds, targets, and pathways, a “drug–active ingredient–target-pathway” was constructed based on the top 10 KEGG pathways (by *P* value). The network was visualized using Cytoscape 3.10.3, and the final network diagrams were exported.

### 2.5. Molecular docking

Based on the degree values, the top 5 key targets from the PPI network and the top 5 active compounds from the “compound–target” network were selected for molecular docking analysis. The 3-dimensional (3D) structures of the selected active compounds were obtained from the PubChem database (https://pubchem.ncbi.nlm.nih.gov/). The ligand structures were optimized using Chem3D software and subsequently converted into accurate 2-dimensional (mol2) formats for docking. The 3D structures of the target proteins were retrieved from the RCSB Protein Data Bank (https://www.rcsb.org/). Water molecules and irrelevant ligands were removed using PyMOL 2.6.0 software (Schrödinger, Inc., New York). Hydrogen atoms were added to the protein structures using AutoDockTools 1.5.7, and the ligands were also hydrogenated with rotatable bonds defined. The docking grid box was centered at *x* = 10.293, *y* = −6.702, *z* = −16.035, with the grid size adjusted according to the volume of the binding pocket. Docking parameters were set as follows: exhaustiveness = 8 (or 20), num_modes = 20, and energy_range = 3. To ensure statistical robustness, each ligand was docked independently 3 times. The binding affinities and orientations of SGML-4 active compounds with their target proteins were then determined. The protein–ligand complexes with the most stable binding conformations were visualized using Discovery Studio 2019 software (Dassault Systèmes BIOVIA, Waltham).

### 2.6. Molecular dynamics simulation

To validate the binding strength and stability of the protein–ligand complexes, we conducted molecular dynamics simulations on the complexes that showed stable hydrogen bonding and high binding affinity, based on the molecular docking results. GROMACS 2024.2 was used to perform 100-nanosecond (ns) MD simulations. Ligand topologies were generated using the Sobtop tool (http://sobereva.com/soft/Sobtop) based on the AMBER GAFF force field, while the protein and ligand parameters were generated using the AMBER14SB force field integrated in GROMACS 2024.2. Periodic boundary conditions were applied to define the simulation box, which was subsequently solvated with water molecules. Sodium (Na⁺) and chloride (Cl−) ions were added at a concentration of 0.15 mol/L to neutralize the system. Energy minimization was performed using the steepest descent algorithm to reduce the system’’s potential energy. The equilibration process was performed in 2 stages: an initial 100 ps NVT ensemble (constant number of particles, volume, and temperature) at 300 K to stabilize the system temperature, followed by a 100 ps NPT ensemble (constant number of particles, pressure, and temperature) at 1 bar to stabilize the system pressure. The simulation results were visualized using DuIvyTools (https://duivytools.readthedocs.io/en/v0.6.0/DIT.html). The root mean square deviation (RMSD), root mean square fluctuation (RMSF), and radius of gyration (Rg) were calculated from the simulation trajectories to assess the structural stability and binding behavior of the protein–ligand complexes.

## 3. Results

### 3.1. Screening of active components and targets of SGML-4

The active components of SGML-4 were identified using the Traditional Chinese Medicine Systems Pharmacology, Herbal Medicine Resource, and Encyclopedia of Traditional Chinese Medicine databases, with additional compounds from Cuminum cyminum (Xianghanqin), which were not available in these databases, and supplemented by a literature review. After screening with SwissADME and removing duplicates, 151 valid active compounds were obtained. After retrieving the compound-related targets through the SwissTargetPrediction platform and selecting those with a probability value >0.1, a total of 106 active compounds and 858 corresponding targets were obtained (all compounds included in the subsequent analyses and their physicochemical properties are listed in Table S1, Supplemental Digital Content, https://links.lww.com/MD/Q873). Based on the constructed “drug–active compound–target” network (Figure S1, Supplemental Digital content, https://links.lww.com/MD/Q871), the top 5 active compounds were identified according to their degree values, namely luteolin, pinocembrin, piplartine, lysicamine, and apigenin (Table 1).

**Table 1:**
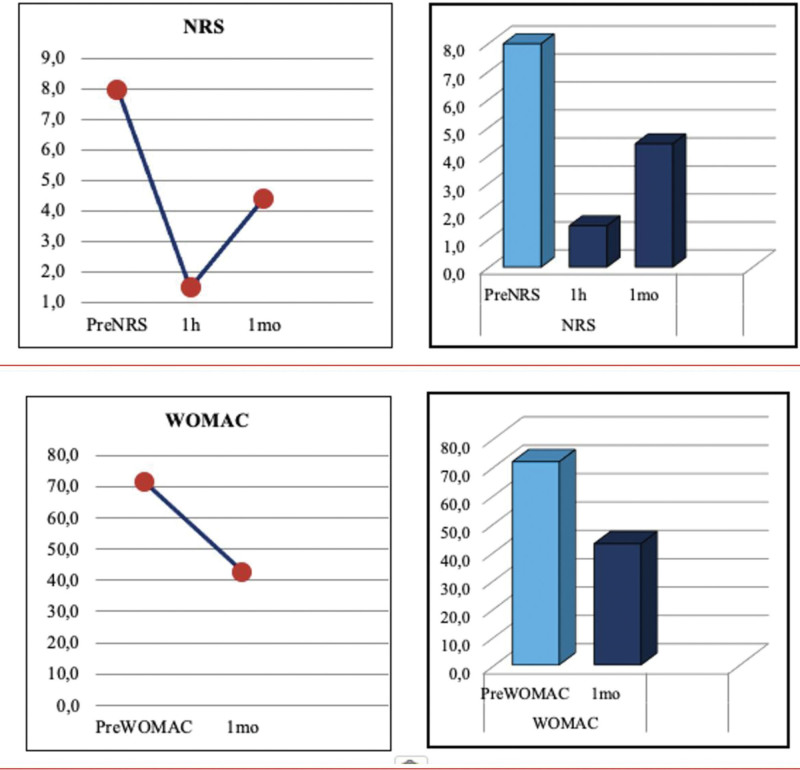
Active compounds and structures.

### 3.2. Identification of insomnia-related targets and prediction of potential anti-insomnia targets of SGML-4

**Figure 1.**
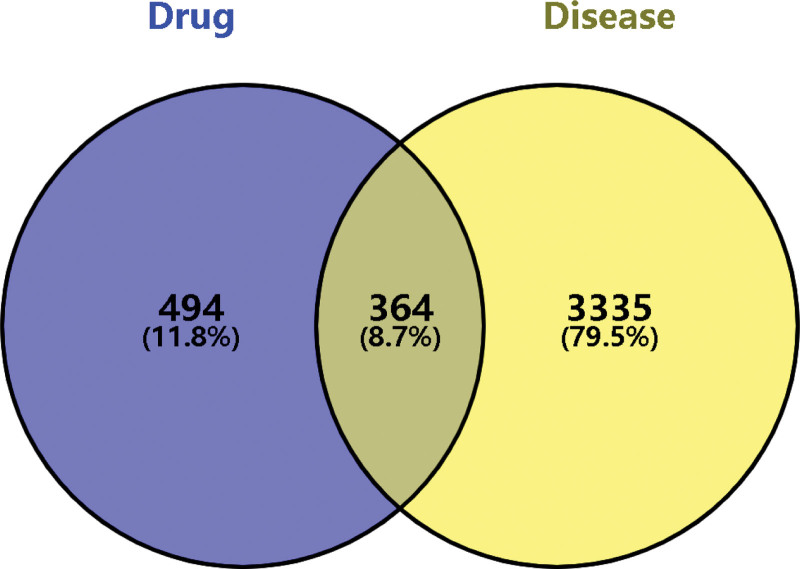
Active ingredient–disease 458 intersecting targets.

Based on the keyword “Insomnia,” we obtained 7035, 36, 27, 416, and 4 disease-related targets from the GeneCards, Online Mendelian Inheritance in Man, Therapeutic Target Database, Drugbank, and PharmGKB databases, respectively. Following standardization and the elimination of duplicate entries, a total of 3847 unique disease-related targets were obtained (Table S2, Supplemental Digital Content, https://links.lww.com/MD/Q873). Venny 2.1 was then used to identify the intersection between the insomnia-related targets and the predicted targets of the active SGML-4 compounds, which resulting in 364 potential therapeutic targets for SGML-4 in the treatment of insomnia (Fig. 1).

### 3.3. Construction of the PPI network

**Figure 2.**
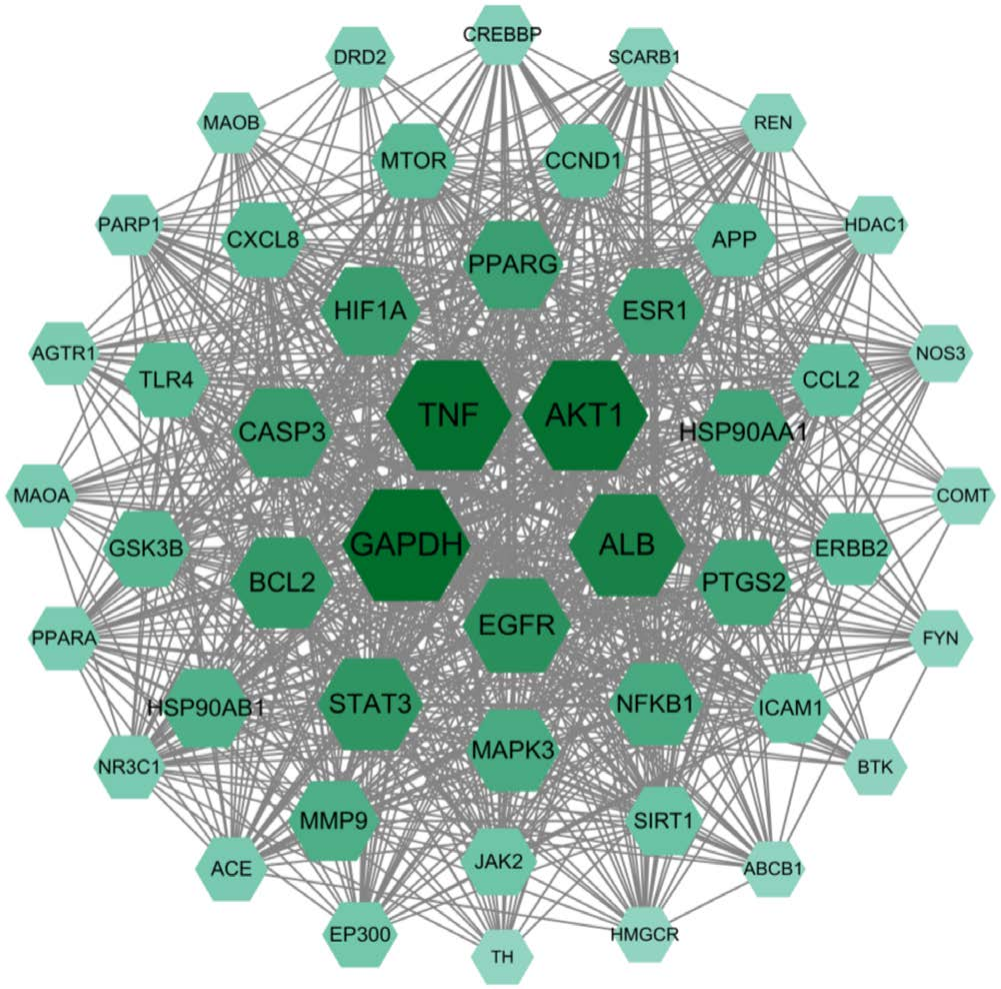
Protein–protein interaction network diagram showing the top 50 targets.

**Figure 3.**
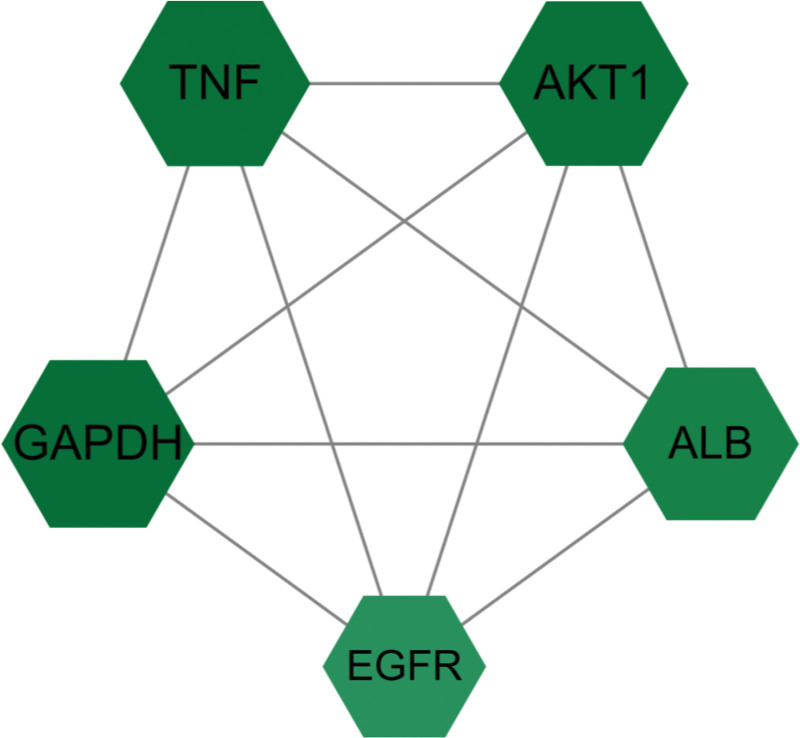
Network diagram of protein–protein interactions between the 5 core targets.

The topological information of the PPI network comprising 364 intersecting targets was retrieved from the STRING database (Figure S2, Supplemental Digital content, https://links.lww.com/MD/Q871 and Table S3, Supplemental Digital Content, https://links.lww.com/MD/Q873). The network was visualized using Cytoscape, with larger node sizes and higher degree values representing stronger protein–protein interactions. Topological analysis of the PPI network was performed using the CytoNCA plugin, and the top 48 targets ranked by the degree value were selected (Fig. 2). Of these, the top 5 targets were selected as core targets for SGML-4 in the treatment of insomnia (Fig. 3). From these results, it is evident that these targets may affect insomnia through inflammation and immune responses (TNF, CXCL8, PTGS2, etc); signal transduction (AKT1, epidermal growth factor receptor [EGFR], STAT3, etc); apoptosis (BCL2, CASP3, etc); metabolism (glyceraldehyde-3-phosphate dehydrogenase [GAPDH], albumin [ALB], PPARG, etc); stress response (HIF1A, HSP90AA1, HSP90AB1, etc); and other (ESR1, APP, etc) physiological processes.

### 3.4. GO Functional and KEGG Pathway Enrichment Analysis

**Figure 4.**
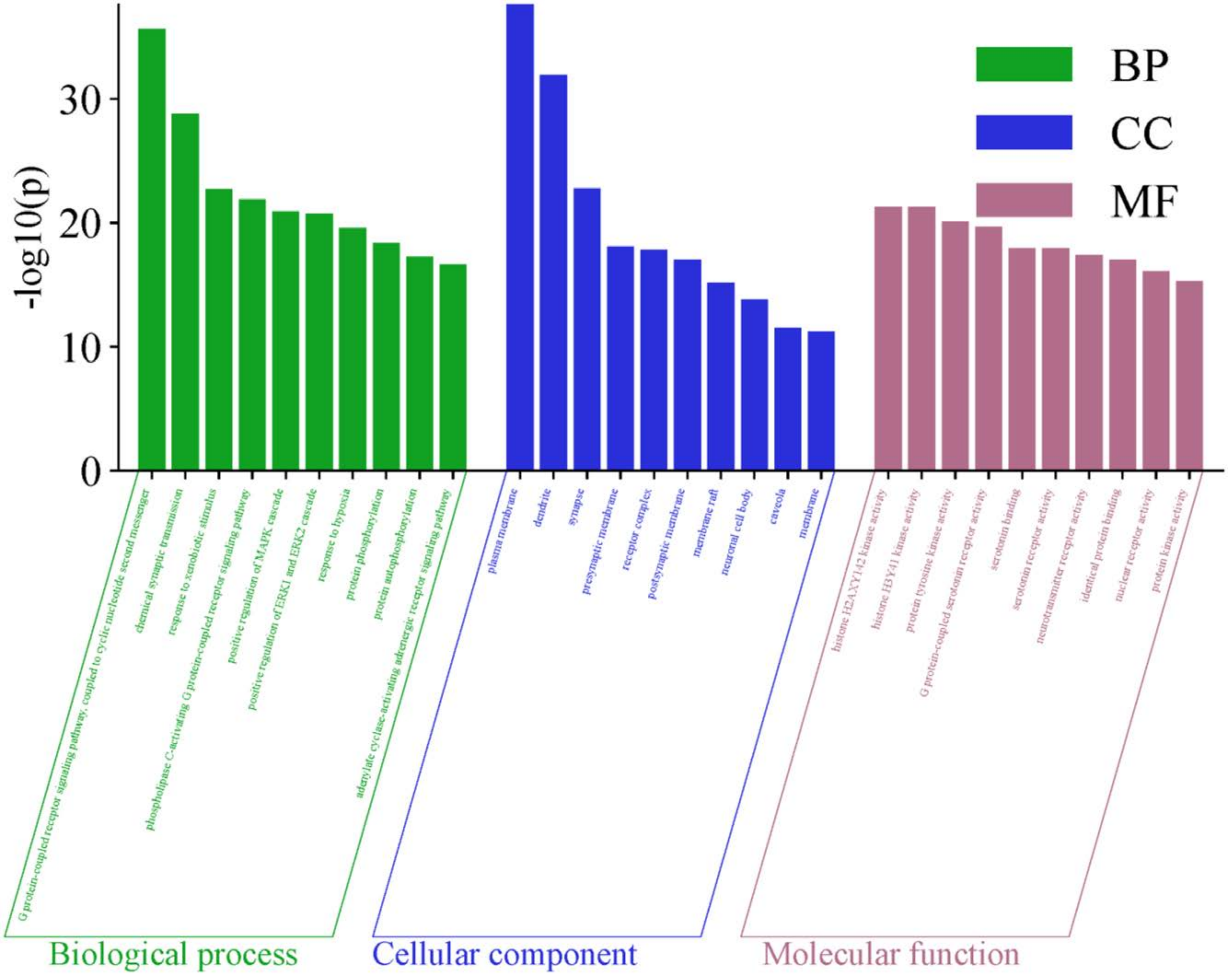
Analysis of the Gene Ontology enrichment results.

**Figure 5.**
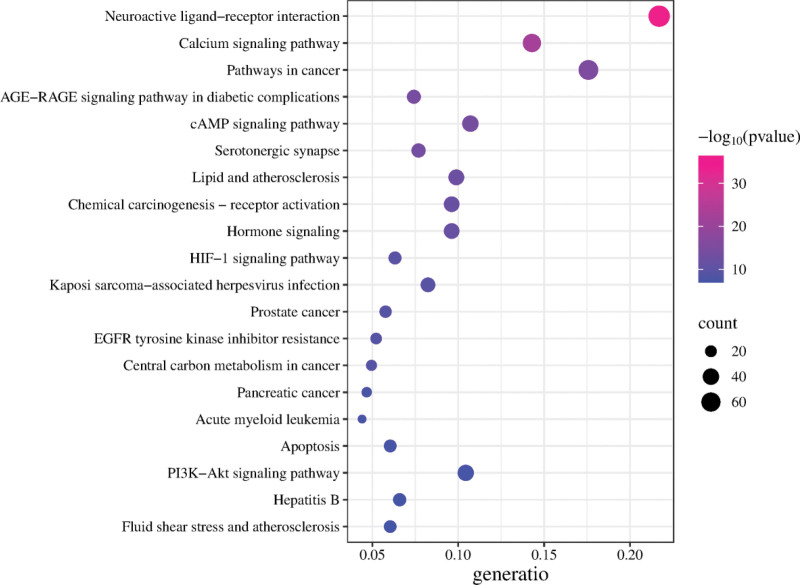
Analysis of the Kyoto Encyclopedia of Genes and Genomes enrichment results.

**Figure 6.**
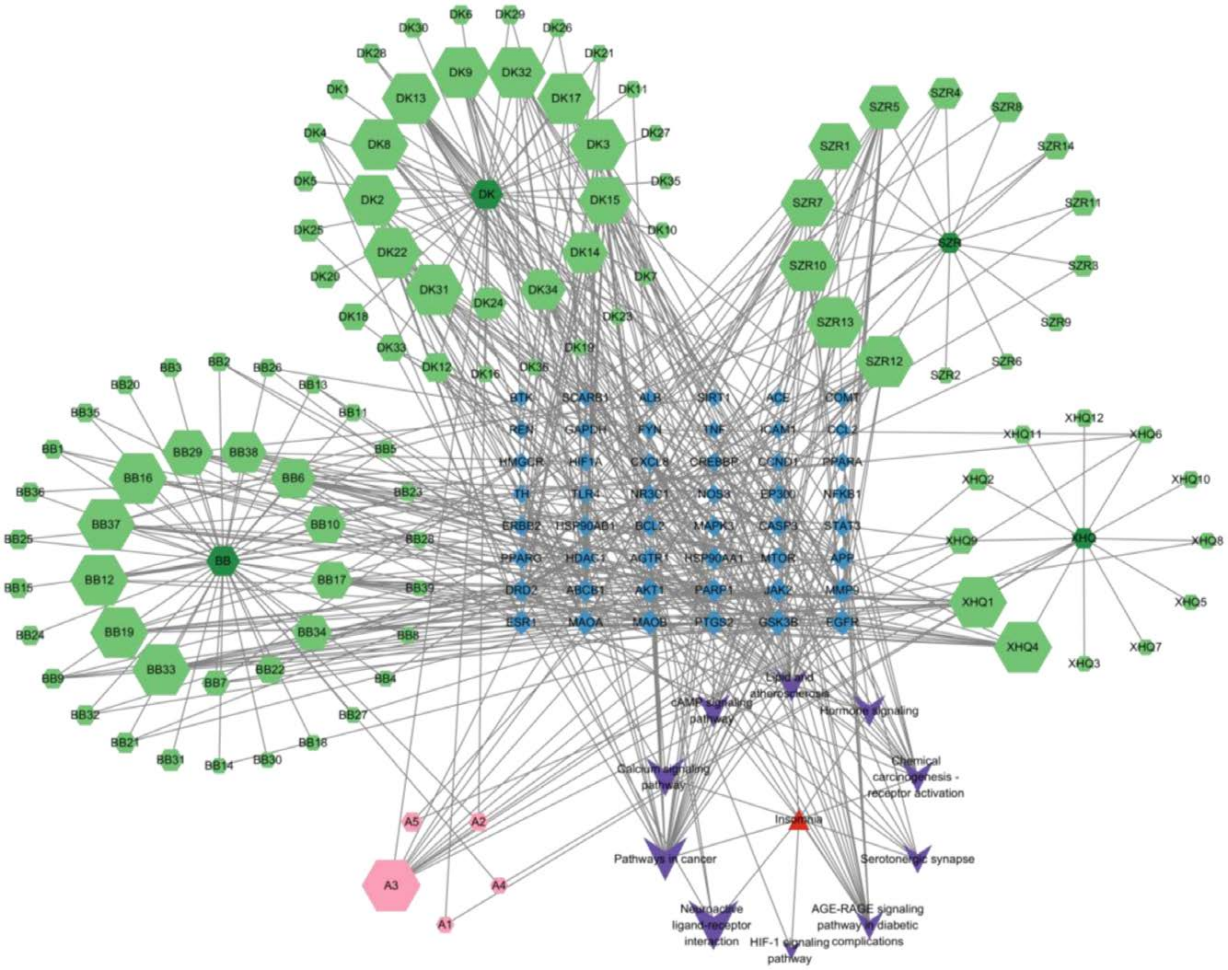
“Herb–active compound–key target-pathway” network. (The dark green hexagon represents the SGML-4 formula; the light green hexagons represent its active compounds; the pink hexagons labeled A1–A5 denote the common active compounds; the blue quadrilaterals indicate the intersecting targets; the red triangles represent the disease; and the purple “V”-shaped symbols indicate the key pathways).

The intersection targets of SGML-4 and insomnia were imported into the DAVID platform for GO and KEGG enrichment analyses. Information regarding BP, CC, MF, and KEGG pathways was collected, with a significance threshold of *P* < .05. A total of 710 BP terms, 112 CC terms, 241 MF terms, and 143 KEGG pathways were identified(Tables S4–S7, Supplemental Digital Content, https://links.lww.com/MD/Q873). The top 10 terms were ranked by *P* value and visualized as bubble plots (Figs. 4 and 5). Biological process analysis revealed that the targets were mainly involved in G protein-coupled receptor signaling, chemical synaptic transmission, responses to exogenous stimuli, and positive regulation of mitogen-activated protein kinase (MAPK) cascade. Cellular component analysis indicated that the targets primarily acted in the plasma membrane, dendrites, postsynaptic membrane, synapse, neuronal cell bodies, and neuronal projections. Molecular function analysis showed enrichment in histone H2AX Y142 kinase activity, protein tyrosine kinase activity, G protein-coupled serotonin receptor activity, serotonin binding, serotonin receptor activity, and neurotransmitter receptor activity. KEGG pathway enrichment analysis highlighted neuroactive ligand–receptor interaction, calcium signaling pathway, cancer-related pathways, AGE–RAGE signaling pathway in diabetic complications, cyclic adenosine monophosphate (cAMP) signaling pathway, and serotonergic synapse, among others. To clearly illustrate the relationships between active compounds, key targets, and signaling pathways, a “drug–active compound–key target-pathway” network was constructed based on the collected data (Fig. 6). In this network, each node is connected to multiple edges, reflecting that the anti-insomnia effect of SGML-4 is not mediated by a single active compound alone, but rather arises from the synergistic interactions of multiple compounds acting on multiple targets and pathways.

### 3.5. Molecular docking

**Figure 7.**
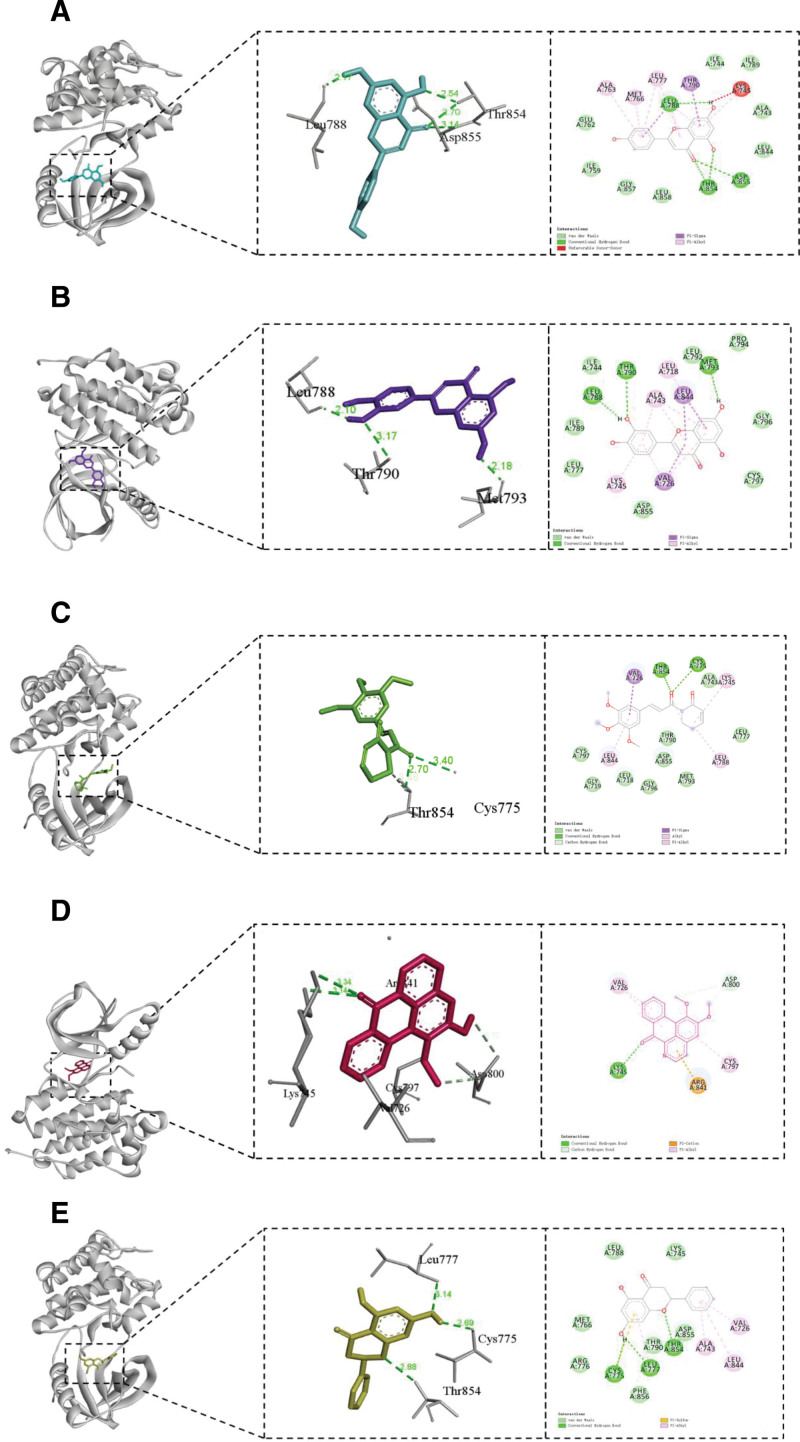
Results of molecular docking of EGFR with 5 active ingredients. (A) Apigenin, (B) luteolin, (C) piplartine, (D) dauricine, and (E) pinocembrin. EGFR = epidermal growth factor receptor.

Based on degree values, the top 5 key targets from the PPI network (AKT1, GAPDH, TNF, ALB, and EGFR) and the top 5 active compounds of SGML-4 (luteolin, pinocembrin, piplartine, lysicamine, and apigenin) were selected for molecular docking. Each ligand–target pair was docked independently 3 times, and the best binding energies are summarized in Table 2 (the standard deviation and range are provided in Table S8, Supplemental Digital Content, https://links.lww.com/MD/Q873). The results indicated that all 5 key targets were capable of binding with the 5 active compounds, with binding energies below −6.0 kcal/mol. Notably, although EGFR did not always achieve the absolute lowest binding energy, it exhibited relatively low and stable binding affinities with multiple compounds (luteolin: −9.8 kcal/mol; apigenin: −9.6 kcal/mol; piplartine: −8.4 kcal/mol), and hydrogen bonds were consistently formed. The docking poses of EGFR are visualized in Figure 7, highlighting its high potential value in SGML-4 compound–target interactions.

**Table 2:** Molecular docking binding energies.

	AKT1	TNF	ALB	EGFR	GAPDH
Luteolin	-10.1	-6.7	-9.3	-9.8	-10.4
Pinocembrin	-9.7	-9.2	-9.6	-9.3	-9.8
Apigenin	-9.7	-9.2	-9.7	-9.6	-8.4
Lysicamine	-10.9	-8.2	-8.0	-7.8	-7.6
Piplartine	-8.5	-8.1	-7.1	-8.4	-8.6

ALB = albumin, EGFR = epidermal growth factor receptor, GAPDH = glyceraldehyde-3-phosphate dehydrogenase, TNF = tumor necrosis factor.

### 3.6. Molecular dynamics simulation

**Figure 8.**
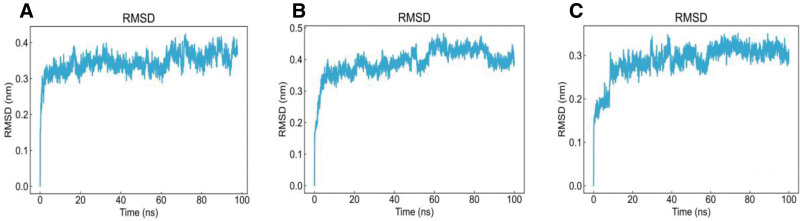
Protein root mean square deviation. (A) EGFR–apigenin, (B) EGFR–luteolin, and (C) EGFR–piplartine. EGFR = epidermal growth factor receptor.

**Figure 9.**
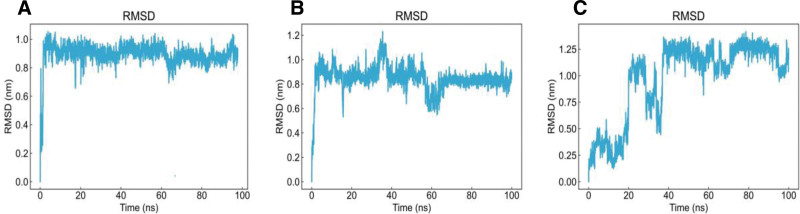
Ligand root mean square deviation. (A) EGFR–apigenin, (B) EGFR–luteolin, and (C) EGFR–piplartine. EGFR = epidermal growth factor receptor.

**Figure 10.**
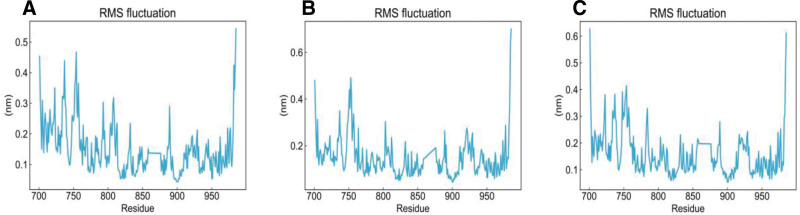
Root mean square fluctuation. (A) EGFR–apigenin, (B) EGFR–luteolin, and (C) EGFR–piplartine. EGFR = epidermal growth factor receptor.

**Figure 11.**
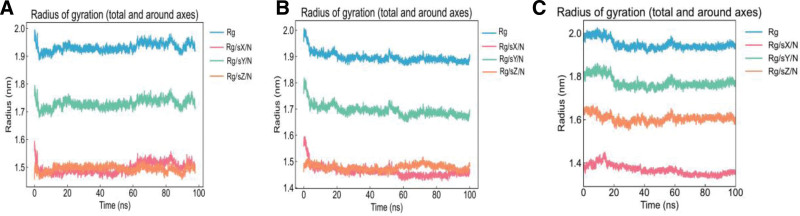
Radius of gyration. (A) EGFR–apigenin, (B) EGFR–luteolin, and (C) EGFR–piplartine. EGFR = epidermal growth factor receptor.

To further evaluate the binding stability and interaction degree between ligands and the protein, molecular dynamics simulations were performed for 100 ns based on the results of network pharmacology and molecular docking. EGFR complexed with luteolin, apigenin, and piplartine was selected for simulation, consistent with the focus of previous studies. The stability of the complexes was assessed by analyzing the RMSD, RMSF, and Rg of the trajectories. During the simulation, the protein maintained relative stability, and Rg values below 2 nm indicated a compact molecular structure. As shown in Figures 8–11, the RMSD curves of EGFR complexed with apigenin, luteolin, and piplartine stabilized after approximately 10 ns, with RMSD values ranging from 0.3 to 0.5 nm. Specifically, the RMSD of apigenin fluctuated between 0.2 to 0.8 nm and tended to stabilize overall; luteolin fluctuated between 0.2 to 1.0 nm, with occasional higher deviations; piplartine exhibited fluctuations between 0.25 to 1.2 nm, larger initially but gradually converging over time. The RMSD fluctuations of the ligands were generally greater than those of the protein backbone, suggesting conformational adjustments within the binding pocket, but all remained below 1.2 nm without evidence of dissociation or destabilization. Apigenin and luteolin exhibited relatively more stable binding, whereas piplartine displayed greater conformational flexibility. The RMSF analysis indicated that most EGFR residues fluctuated <0.5 nm during the simulation, suggesting relative stability of amino acid residues. The Rg values of the protein remained constant and below 2 nm throughout the simulation, confirming the compactness of the protein structure. Overall, the molecular dynamics simulation results demonstrated stable interactions between EGFR and the 3 active compounds, further validating the reliability of the molecular docking predictions. These findings provide valuable insights into how these compounds may modulate target protein activity and contribute to therapeutic effects.

## 4. Discussion

In this study, we used a network pharmacology approach to predict the potential targets of SGML-4 for the treatment of insomnia.

(1) As a traditional Mongolian medicine, SGML-4 contains multiple active compounds. Based on the results of this study, luteolin, pinocembrin, piplartine, nantenine, and apigenin are considered to be the key potential active compounds contributing to its therapeutic effects. Luteolin, a natural flavonoid, has been shown to promote sleep by activating the adenosine A1R and A2AR receptors (A1R and A2AR). The adenosine system plays a central role in sleep homeostasis; A1R inhibits arousal-related neurons, and A2AR enhances sleep drive, thereby synergistically regulating non-rapid eye movement sleep. Unlike conventional benzodiazepine sedatives, luteolin not only prolongs sleep duration but also deepens sleep, potentially improving overall sleep quality.^[46]^ Luteolin also improves anxiety and depression-like behaviors induced by sleep deprivation through the inhibition of the NF-κB/NLRP3 inflammasome pathway in the hippocampus; this mechanism is linked to the suppression of inflammatory cytokines and restoration of neuronal plasticity.^[47]^ Pinocembrin, also known as pinobanksin, is another natural flavonoid that exerts neuroprotective effects against intermittent hypoxia-induced neuroinflammation. It suppresses NLRP3 inflammasome activation through BNIP3-dependent mitophagy, thereby improving cognitive function and reducing neuroinflammation.^[48]^ Lysicamine, a natural compound belonging to the isoquinoline alkaloid class, has been reported to possess antibacterial, antiinflammatory, and antitumor activities.^[49,50]^ Piplartine, also known as Piperlongumine, is an alkaloid compound. It has various biological activities, including antitumor, anti-inflammatory, analgesic, antifungal, anti-schistosomal, anxiolytic, and antidepressant effects.^[51–53]^ Apigenin, also known as apiumin or apigenol, is a plant-derived flavonoid. It promotes GABA synthesis through the activation of chloride channels and upregulation of generalized anxiety disorder, which synergizes with pentobarbital to enhance inhibitory synaptic transmission and prolong sleep duration.^[54]^ Its antidepressant and anxiolytic effects are associated with the suppression of inflammatory markers (e.g., TNF-α, IL-6, IL-1β); inhibition of NF-κB activation; modulation of monoamine neurotransmitters (dopamine, serotonin); normalization of hypothalamic–pituitary–adrenal axis dysfunction; and downregulation of the cAMP signaling pathway.^[55]^ Notably, among the 5 potential active compounds, luteolin, and apigenin primarily act directly on the nervous system, with luteolin modulating sleep-related pathways and apigenin enhancing inhibitory neural signaling, while also exhibiting indirect neuroprotective effects, such as anti-inflammatory activity. Piplartine exerts direct modulatory effects on the nervous system, including anxiolytic and antidepressant actions. In contrast, pinocembrin and lysicamine primarily display direct non-neuronal effects, such as anti-inflammatory, antitumor, and antibacterial activities, and have not yet been shown to exert explicit direct regulation of the nervous system.
(2) SGML-4 exhibits multiple core targets, with the top 5 based on degree values being EGFR, AKT1, GAPDH, TNF, and ALB. EGFR, a member of the receptor tyrosine kinase superfamily, plays a critical role in cell growth, development, and proliferation. In the suprachiasmatic nucleus (SCN), EGFR shows pronounced circadian transcriptional responses and regulates rhythm-related pathways through transcription factors such as CREB, AP1, and Rorα, providing a molecular basis for the SCN to integrate environmental signals with the intrinsic biological clock.^[56]^ Dysregulation of this mechanism may lead to circadian rhythm disturbances and insomnia. Moreover, EGFR signaling regulates sleep through the MAPK/ERK–NPVF pathway^[57]^ and plays an important role in the sleep–wake cycle.^[58,59]^ RAC-alpha serine/threonine–protein kinase (AKT1) is a key mediator in cellular signal transduction and can reverse lipopolysaccharide-induced sleep–wake disturbances by activating orexin neurons.^[60]^ TNF is a multifunctional cytokine that serves as both a potential therapeutic target in cancer and an intervention point in inflammatory diseases.^[61]^ GAPDH, besides its role in glycolysis, regulates transcriptional activation and apoptosis.^[62]^ ALB, the major plasma protein, shows an inverted U-shaped relationship with sleep duration in American adults, peaking at approximately 7.5 hours of sleep, with significantly lower levels observed with shorter or longer sleep.^[63]^ Notably, among these 5 targets, EGFR, AKT1, and ALB are more closely associated with insomnia. EGFR is directly and multi-dimensionally linked to insomnia through regulation of the SCN circadian rhythm and the MAPK/ERK–NPVF sleep pathway. AKT1 is directly related to insomnia via correction of sleep–wake disturbances, while ALB indirectly reflects sleep status through its level fluctuations. Among the 3, EGFR shows the strongest association with insomnia, as it is involved in both the “core circadian clock” and the “sleep–wake cycle.”
(3) The functional enrichment analysis indicated that the occurrence of insomnia may involve multiple key pathways and molecular mechanisms. G protein-coupled receptors play a central role in regulating neurotransmitter activity,^[64]^ particularly 5-HT receptors,^[65]^ which are considered important targets for insomnia drug development due to their close involvement in mood, anxiety, and sleep regulation. Serotonin receptor activity is closely associated with sleep maintenance, and subtypes such as 5-HT1A and 5-HT2A^[66]^ serve as key targets for modulating mood and promoting sleep. Additionally, synaptic transmission and synapse-related pathways (including chemical synaptic transmission, pre- and postsynaptic membranes, dendrites, and neuronal cell bodies) are closely related to insomnia, supporting the view that insomnia is associated with an imbalance between excitatory (glutamate) and inhibitory (GABA) neurotransmitters.^[67]^ These findings suggest that the targets identified in this study may exert regulatory effects in the central nervous system through multi-target modulation of neurotransmitter signaling, synaptic structure, and function, thereby contributing to sleep improvement. This provides a theoretical basis and directional guidance for subsequent mechanistic validation and pharmacological experiments. Among the KEGG-enriched pathways, the serotonergic synapse, which primarily utilizes 5-HT as a neurotransmitter, can improve fatigue in sleep-deprived mice through regulation of 5-HT levels.^[68]^ The synthesis and release of 5-HT are influenced by light and circadian rhythms, thereby affecting melatonin production and the sleep–wake cycle.^[69]^ The cAMP signaling pathway transmits signals by regulating intracellular second messenger cAMP concentrations, which in turn affect transcription factors such as CREB and regulate circadian gene expression.^[70]^ The calcium signaling pathway involves intracellular Ca²⁺ fluctuations that trigger downstream signaling; studies have shown that total sleep deprivation reduces hippocampal Ca²⁺ influx, impairing neuronal plasticity and leading to cognitive deficits.^[71]^ The enriched pathways identified in this study (including serotonergic synapse, cAMP signaling, and calcium signaling) are consistent with previous network pharmacology studies on herbal treatments for insomnia,^[72,73]^ indicating their common involvement in insomnia pathogenesis. Notably, this study also identified significant enrichment of the HIF-1 signaling pathway and apoptosis-related genes, which have rarely been reported in other herbal insomnia network pharmacology studies, suggesting that hypoxic stress and cell apoptosis may represent novel mechanisms influencing insomnia.
(4) Molecular docking results indicated that the top 5 active compounds exhibited favorable binding affinities with the target proteins. Focusing on the docking results of EGFR with luteolin, apigenin, and piplartine, all 3 formed relatively stable complexes with EGFR. The binding energies were − 9.8 kcal/mol for luteolin, −9.6 kcal/mol for apigenin, and − 8.4 kcal/mol for piplartine. According to the core criterion in molecular docking, “the more negative the binding energy, the stronger the compound–protein affinity and the more stable the binding,” a gradient in binding strength among the 3 compounds can be observed. Luteolin and apigenin both had binding energies approaching − 10.0 kcal/mol, indicating strong affinity for EGFR, with only a 0.2 kcal/mol difference between them, suggesting comparable binding strength. Piplartine, although exhibiting a higher binding energy than the other 2, remained below − 8.0 kcal/mol, indicating it can also form a stable complex with EGFR, albeit with relatively weaker affinity. For comparison, the reported binding energy range of commonly used insomnia drugs, such as zolpidem with the GABA_A receptor, is approximately − 7.0 to − 9.0 kcal/mol.^[74]^ Notably, some SGML-4 components exhibit binding energies to EGFR that are comparable or slightly stronger, highlighting that luteolin, apigenin, and piplartine (with their direct neuromodulatory effects) possess a solid molecular basis as potential EGFR ligands most relevant to insomnia. Among these, luteolin and apigenin, due to their stronger EGFR affinity, may warrant further in-depth investigation in studies exploring EGFR-related mechanisms.
(5) Molecular dynamics simulation results demonstrated that the primary active compounds (luteolin, apigenin, and piplartine) formed stable complexes with the core target EGFR. These findings further confirm the central role of luteolin, apigenin, and piplartine in mediating the anti-insomnia effects of SGML-4 via EGFR. It is worth noting that, although this study focused on EGFR, insomnia involves multiple signaling pathways, such as GABAergic metabolism, serotonergic signaling, and melatonin secretion regulation. EGFR may interact with these classical pathways to participate in sleep regulation, which warrants further investigation. In addition, only a single 100 ns simulation of each complex was performed in this study. Although the system reached structural stability around 50 ns (RMSD convergence and minimal energy fluctuations), a single trajectory has inherent limitations in statistical robustness. Therefore, future studies should employ multiple independent trajectories to enhance the reliability and reproducibility of the results.

In summary, this study employed network pharmacology, molecular docking, and molecular dynamics simulations to explore the potential mechanisms by which SGML-4 exerts its anti-insomnia effects through a multi-component, multi-target, and multi-pathway approach. Our results suggest that apigenin, luteolin, and piplartine are the key potential active compounds in SGML-4 for the treatment of insomnia. Notably, EGFR appears to be a critical target for all 3 compounds, potentially mediating the therapeutic effects of SGML-4 via pathways such as serotonergic synapses, calcium signaling, and cAMP signaling. It should be noted that this study is entirely based on computational analyses using public databases, which cannot directly demonstrate therapeutic efficacy. Moreover, each database emphasizes different aspects, introducing certain limitations to our study. Considering the complexity of the components and mechanisms of Mongolian medicine formulations, further experimental validation of SGML-4’s anti-insomnia effects in cellular and animal models is warranted, with the aim of providing a scientific basis for its clinical application in the treatment of insomnia.

## Acknowledgments

Both Dr Chula Sa and Dr Lechaolu Bao contributed equally to this work and should be considered co-corresponding authors.

## Author contributions

**Conceptualization**: Rina Su, Chula Sa, Lechaolu Bao.

**Data curation**: Rina Su, Chula Sa.

**Formal analysis**: Rina Su, Dena Su, Riguga Su.

**Investigation**: Rina Su, Jinjing Wang.

**Methodology**: Rina Su, Jinjing Wang, Riguga Su, Chula Sa.

**Project administration**: Lechaolu Bao.

**Resources**: Lechaolu Bao.

**Supervision**: Chula Sa, Lechaolu Bao.

**Software**: Rina Su, Dena Su.

**Validation**: Rina Su, Dena Su, Riguga Su.

**Visualization**: Rina Su, Jinjing Wang.

**Writing – original draft**: Rina Su.

**Writing – review & editing**: Chula Sa, Lechaolu Bao.

## Supplementary Material
