# Supplementary material for: Prediction of the therapeutic mechanism of Sugemule-4 in insomnia treatment using network pharmacology and molecular docking
Source: Medicine (Baltimore). 2026 May 12;104(49):e46489. doi: 10.1097/MD.0000000000046489 (PMC12688716; doi:10.1097/MD.0000000000046489)

Fig. S1. “Drug–Active Compound–Target” Network. The orange nodes represent the drugs, the peripheral pink nodes indicate the drug components, the central green nodes correspond to the component-related targets, and the purple nodes represent the common components among the drugs. The larger the node and the higher the degree value, the stronger the interaction.


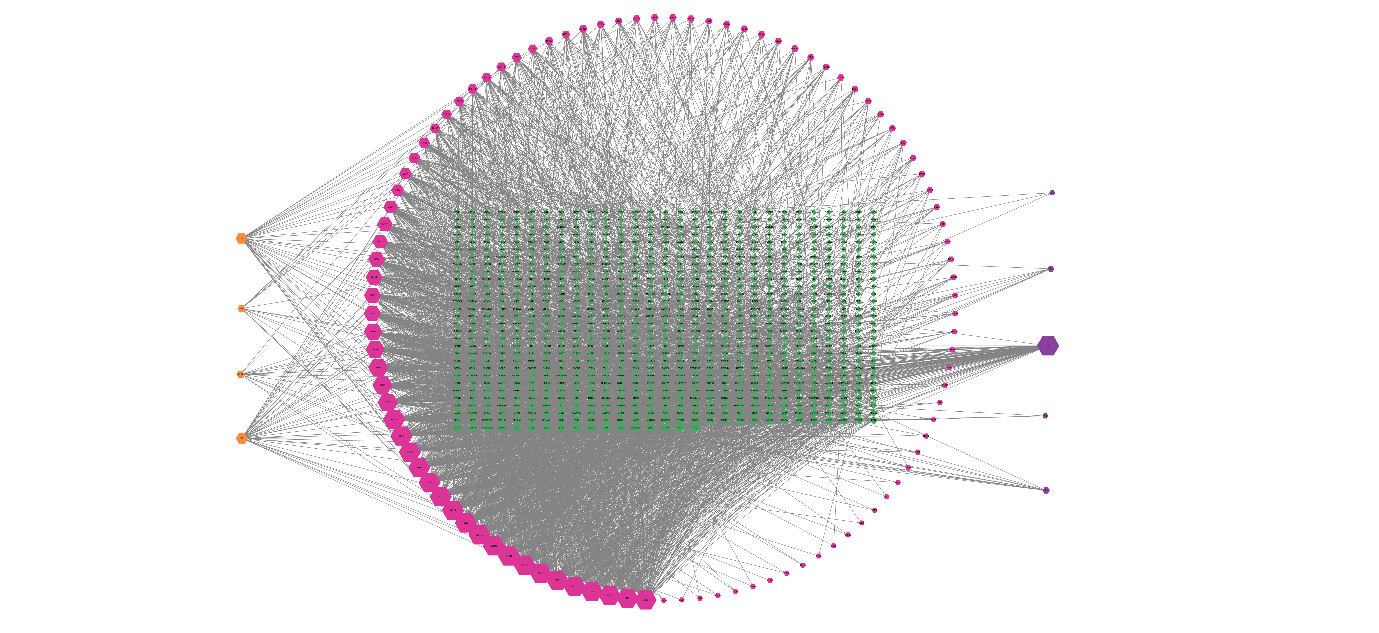


Fig. S2. Protein–protein interaction (PPI) network of 364 intersecting targets. A total of 364 intersecting targets were analyzed using the STRING database, and the resulting PPI network was visualized with Cytoscape software. Each node represents a protein, and edges indicate interactions between them. The size and color depth of the nodes correspond to the degree value, with larger and darker nodes indicating higher connectivity.


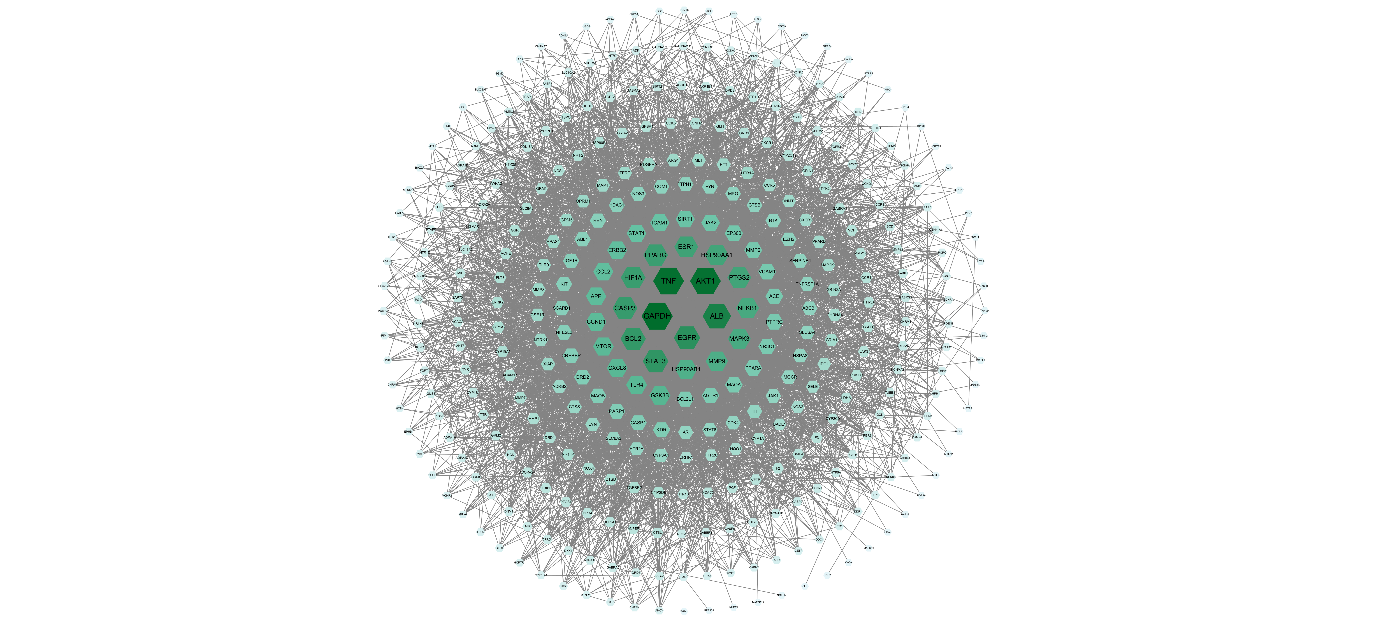

Supplement: Supplementary file 1 [file medi-104-e46489-s001.docx]
